# Clinical and pathological significance of *Homo sapiens* ceramide synthase 2 (CerS-2) in diverse human cancers

**DOI:** 10.1042/BSR20181743

**Published:** 2019-05-07

**Authors:** Qian Zhang, Jin-yan Wang, Wei Yan, Dan-dan Wang, Su-jin Yang, Si-ying Zhou, Shan-liang Zhong, Jin-hai Tang

**Affiliations:** 1 Department of General Surgery, The Affiliated Cancer Hospital of Nanjing Medical University and Jiangsu Cancer Hospital and Jiangsu Institute of Cancer Research, Nanjing 210029, P.R. China; 2 Center of Clinical Laboratory Science, Jiangsu Cancer Hospital and Jiangsu Institute of Cancer Research and The Affiliated Cancer Hospital of Nanjing Medical University, Baiziting 42, Nanjing 210029, P.R. China; 3 The First Clinical Medical College, Nanjing University of Chinese Medicine, Nanjing 210023, P.R. China; 4 Department of General Surgery, The First Afﬁliated Hospital of Nanjing Medical University, Nanjing 211166, P.R. China

**Keywords:** Cancers, Clinical and pathological significance, Homo sapiens ceramide synthase 2

## Abstract

*Homo sapiens* ceramide synthase 2 (CerS-2) plays an important role in inhibiting invasion and metastasis of tumor cells and has been reported as a tumor metastasis suppressor gene in diverse cancers. Thus, low level of CerS-2 protein might suggest a bad prognosis and up-regulation of CerS-2 protein might act as a promising therapeutic strategy for malignant tumors. In this review, we discussed the expression, as well as the clinical and pathological significance of CerS-2 in diverse human cancers. The pathological processes and molecular pathways regulated by CerS-2 were also summarized.

## Introduction

Cancer is a major public health problem worldwide and represents a huge group of complex and multifactorial diseases that involve abnormal cell growth with the potential to invade adjacent and distant tissues. There is a large number of treatments directed against different cancers, but cancer is still the leading cause of death all round the world. Metastasis is the end result of tumor progression and the most common cause of mortality in cancer patients [1]. The progression of primary tumors to metastatic disease refers to the following directions: invasion of extracellular matrix and stromal layers by the tumor cells, intravasation into the bloodstream to travel to a distant organ, extravasation into the parenchyma of distant tissues, and colonization and outgrowth of tumors in the distant site.

Recent studies about diverse cancers have indicated the importance of *Homo sapiens* ceramide synthase 2 (CerS-2) as a tumor-suppressor gene. CerS-2 (also known as L3, LASS2, SP260, and TMSG1), a member of CERS family, was first cloned in 2001 [2]. CerS-2 has a compact gene size, a low number of introns, short 5’- and 3’-UTRs, with a high percentage of surrounding chromosomal sequences containing CpG and Alu elements, and contains a low percentage of LINE-1s. Further, CerS-2 is located within chromosomal regions that are replicated early within the cell cycle [3–5]. What’s more, CerS-2 is found in at least 12 human tissues, with high expression in kidney and liver, moderate expression in brain, heart, skeletal muscle, placenta, and lung, and low expression in colon, thymus, spleen, small intestine, and peripheral blood leukocytes [6]. However, the molecular mechanism underlying CerS-2-mediated inhibition of tumor invasion and metastasis in cancers remains unclear. In this review, we will investigate the clinical and pathological role of CerS-2 in human malignant tumors. Moreover, we will discuss whether CerS-2 can be used as a potential predictive biomarker for cancers and a target for further cancer treatment. Collectively, the information compile here should serve as a comprehensive repository of the current knowledge in this area, and would aid in the design for further studies about CerS-2 in cancers. This review may promote the research and application of CerS-2 for the treatment of cancers.

## Bladder cancer (BC)

Bladder carcinoma (BC) is one category of common genitourinary cancers. Despite significant advances in surgical techniques and adjuvant chemotherapy, BC remains a highly prevalent and lethal malignancy. Studies have shown that rate of bladder carcinoma recurrence in one side of the upper urinary tract cancer was 15–50% or even up to 70% [7]. In recent studies, increased attention has been paid to the association between CerS-2 and bladder carcinoma.

A recent study found that the expression level of CerS-2 in bladder cancer samples was significantly lower than that in the corresponding normal tissues. Remarkably, expression of CerS-2 mRNA was significantly correlated with clinical stage, depth of tumor invasion and recurrence. Patients with CerS-2-negative carcinomas has significantly poorer survival than those with CerS-2-positive carcinomas [8].

CerS-2 was demonstrated to be a direct target of miR-9, which is an up-regulated miRNA in BC. MiR-9 was showed to regulate proliferation, invasion, and chemoresistance of BC cells. MiR-9 could inhibit cisplatin-induced apoptosis and up-regulate the expression of Bcl-2 and survivin by targeting CerS-2, thus promoting chemoresistance of BC cells [9].

Notably, overexpressed miR-93 significantly suppressed CerS-2 protein expression. MiR-93 is a critical effector of BC chemotherapy. Both *in vitro* and *in vivo* experiments demonstrated that the suppression of miR-93 could enhance the chemosensitivity of BC by promoting the expression of CerS-2. But down-regulation of miR-93 had a very slight effect on sensitivity of BC cells to cisplatin when CerS-2 was silenced [10].

A latest study found that miR-3658 can enhance the proliferation, migration and invasion of bladder cancer cells, inhibit cell adhesion, reduce cell chemosensitivity and promote the epithelial–mesenchymal transition of bladder cancer cells through affecting the expression of CerS-2, which again indicates that CerS-2 may be a useful target for further cancer treatment [11].

What’s more, it has been confirmed that CerS-2 was able to regulate V-ATPase activity and pHi through directly interacting with the C subunit of V–ATPase to influence the apoptosis and proliferation of BC cells [12]. The effect of CerS-2 on BC invasion and chemoresistance is partly dependent on Drp1 signaling. CerS-2 can inhibit Drp1 signaling through regulation of ERK pathway [13]. Two genes closely related to cancer metastasis, called MMP-2 and MMP-9, had higher activities in BC cells knocked down for CerS-2 [12]. Thus, it suggested that CerS-2 expression may be correlated with the development and progression of human BC and may be a potential prognostic indicator for this cancer.

## Hepatocellular carcinoma (HCC)

Hepatocellular carcinoma (HCC) is the fifth most common cause of cancer and the most common type of hepatobiliary cancer with over 500,000 new cases diagnosed yearly and has an annual death rate of 2,50,000 people [14], and it’s the third major cause of cancer-related death worldwide [15]. The molecular pathogenesis of HCC is very complex comprising of multiple genetic and epigenetic alterations, chromosomal aberrations, gene mutations, and altered molecular pathways [16].

Despite many improvements have been achieved in clinical treatments such as liver surgical resection, chemotherapy and transplantation, HCC patients still have high recurrence incidence and poor prognosis. Although several molecular biomarkers have been reported to have clinical significance for predicting HCC prognosis [17–19], clinical and molecular features used as prognostic parameters in clinical practice are still substantially needed. Studies have found that CerS-2 was reduced in human HCC at the mRNA and protein levels compared with those in the normal liver [20,21]. As it happens, one study demonstrated that high expression level of CerS-2 and TGF-β 1 has a positive relationship with prognosis of HCC patients. The combination of CerS-2 and TGF-β1 is more sensitive than either of them alone to predict overall survival and time to recurrence [22]. Another experiment showed that CerS-2 is a protective gene against DEN-induced liver carcinogenesis, and deletion of CerS-2 in hepatocytes results in the earlier occurrence of DEN-induced tumors and more rapid tumor growth *in vivo* [23]. These all indicate that CerS-2 could be used as a new independent prognostic marker of HCC patients.

The mechanism by which CerS-2 suppresses HCC may rely upon several factors, one mechanism is related to up-regulation of the TGF-β1–Smad4–PAI-1 axis. Deletion of CerS-2 caused increases in TGF-β1, Smad-4 and -7, which were parallel to the levels of PAI-1 [23]. PAI-1 is a tumor-promoting gene predominantly produced by hepatocytes in liver tissue [24]. TGF-β1 is an effective inducer of PAI-1 expression and the Smad pathway mediates the induction of PAI-1 by TGF-β [25,26]. Another mechanism by which CerS-2 suppresses HCC is via the interaction with miR-694. MiR-694 functions in maintaining homeostasis of the liver, CerS-2 deficiency caused the down-regulation of miR-694 and the up-regulation of its target gene Tnfaip3, which may be related to a high risk of occurrence of HCC [27]. The third mechanism is via the interaction with the V-ATPase. The activity of V-ATPase can be inhibited by ASGR1 through interacting with CerS-2, thereby suppressing the metastatic potential of hepatoma cells [28].

## Breast cancer (BCa)

Breast cancer (BCa) is the most frequently diagnosed female cancer with diverse tumor heterogeneity, which challenges conventional approaches to develop biomarkers for early detection and prognosis [29]. Expression of CerS-2 mRNA was demonstrated to be reduced in breast cancer [30,31], and higher expression of CerS-2 in the breast cancer patients was associated with fewer lymph node metastases [32], and low expression of LASS2 was associated with poor prognosis in patients with BCa [33], thus it could become a candidate marker for the detection and prognosis of breast cancer.

It was found that CerS-2 is heterogeneously expressed in various breast cancer cells. The mRNA and protein expression levels of CerS-2 in poorly invasive breast cancer cells were obviously higher than that in the highly invasive cells. Overexpression of CerS-2 can significantly inhibit the migration and invasion ability of breast cancer cells, whereas knockdown CerS-2 can significantly increase the migration and invasion ability. The mechanism underlying the effects of CerS-2 on breast cancer cells was associated with the decrease of the V-ATPase activity and extracellular hydrogen ion concentration [34], which can cause the inactivation of secreted MMP-2 and non-degradation of ECM [35,36], and ultimately accelerate the breast tumor’s invasion and metastasis.

Resistance to chemotherapy is a main obstacle to overcome during the treatment of tumors. It was found that CerS-2 may enhance the chemosensitivity of breast cancer cells by inhibiting the V-ATPase activity through binding to ATP6V0C. Moreover, a nude mice model showed the combination of CerS-2 overexpression and Dox treatment caused significant decreases in tumor size and weight with respect to controls [33].

Similarly, AGPAT9 was showed to inhibit proliferation, migration, and invasion in breast cancer by inhibiting the V-ATPase activity through up-regulating the mRNA and protein levels of CerS-2 [37]. These all suggest that CerS-2 may be an important influential factor of anti-breast tumor therapeutic efficacy.

## Prostatic cancer (PC)

Prostate cancer (PC) represents the most common cancer and the second most common fatal cancer in men worldwide at present. Specifically, it is evaluated that prostate cancer accounts for ∼33% (233,000) of new cancer cases, and ∼29,480 Americans may die from prostate cancer every year [38]. Factors like older age, family history, and black race can increase the risk of prostate cancer [39]. The expression levels of CerS-2 were lower in the highly metastatic prostatic cell lines, compared with the non-metastatic cell lines [40], which indicate cers2 expression levels were inversely correlated with tumor metastatic potential.

Studies found that silencing of CerS-2 gene can promote growth, proliferation, invasion, and metastasis of human PC cell both *in vitro* and *in vivo*, and CerS-2 had a low expression level in the highly metastatic prostate cancer cell line PC-3M-1E8 [40,41]. Further studies indicated CerS-2 knockdown could enhance the activity of V-ATPase and increase extracellular H+ concentration in PC-3M-2B4 cells, thus inhibiting MMP-2 and MMP-9 activation, and eventually leading to apoptosis of tumor cells [42–44]. Also, tumors with down-regulation of CerS-2 tend to show more frequent lymph node metastasis, indicating that CerS-2 is a novel tumor metastasis suppressor gene for prostate cancer.

One analysis showed that a region (+59 to +123 bp) of exon 1 exhibited a strong role in the initiation of CerS-2 gene transcription, transcription factors KLF6 and Sp1 were able to interact with each other and bound to their elements in this region in human PC cells. Analysis of metastatic capacity showed that cells with high metastatic capability exhibited a lower level of KLF6/TMSG-1 protein and vice versa. Thus, interaction of KLF6 and Sp1, together with their binding of elements in exon 1, are critical events in the initiation of transcription of the CerS-2 gene in human prostate carcinoma cells. This indicates that exon 1 of the CerS-2 gene is not only a structural fragment participating in transcription, but also a part of the regulatory machinery of the gene [45].

## Cervical cancer

Cervical cancer is one of the most common gynecologic malignancies, with approximately 500,000 new cases worldwide and approximately 300,000 deaths each year. In recent years, related reports had shown that the pick incidence of cervical cancer is moving to younger age [46], thus finding an effective way to diagnose and treat cervical cancer is urgent.

One study found that ionizing radiation (IR) induced *de novo* synthesis of ceramide to influence HeLa cell apoptosis by specifically activating CerS-2, CerS-5, and CerS-6 that generate opposing anti- and pro-apoptotic ceramides in mitochondrial membranes. While CerS-2 is responsible for all IR-induced lignoceroyl-CoA-dependent CerS activity in HeLa cells and overexpression of CerS-2 resulted in partial protection from IR-induced apoptosis [47]. This means that CerS-2 plays an important role in radiotherapy of cervical cancer.

## Pheochromocytoma (PCC)

Pheochromocytomas (PCC) are rare, catecholamine-secreting, vascular, neuroendocrine tumors arising from chromaffin cells of the adrenal medulla [48], and for which mutations in 15 disease-associated genes have been identified [49].

A study invested the development of pheochromocytoma in ceramide synthase 2 null mice, and found that ceroid can accumulate rapidly in CerS-2 null mouse adrenal gland, and also mitochondrial become nonfunctional in drenal gland. What’s more, CerS-2 null mice can develop PCC more easily and secrete elevated levels of epinephrine and norepinephrine. This analysis of the role of CerS-2 in PCC may lead to further understanding of the mechanism of development of PCC and may implicate the sphingolipid pathway as a possible novel therapeutic target for this rare tumor [49].

## Conclusions

**Figure 1:**
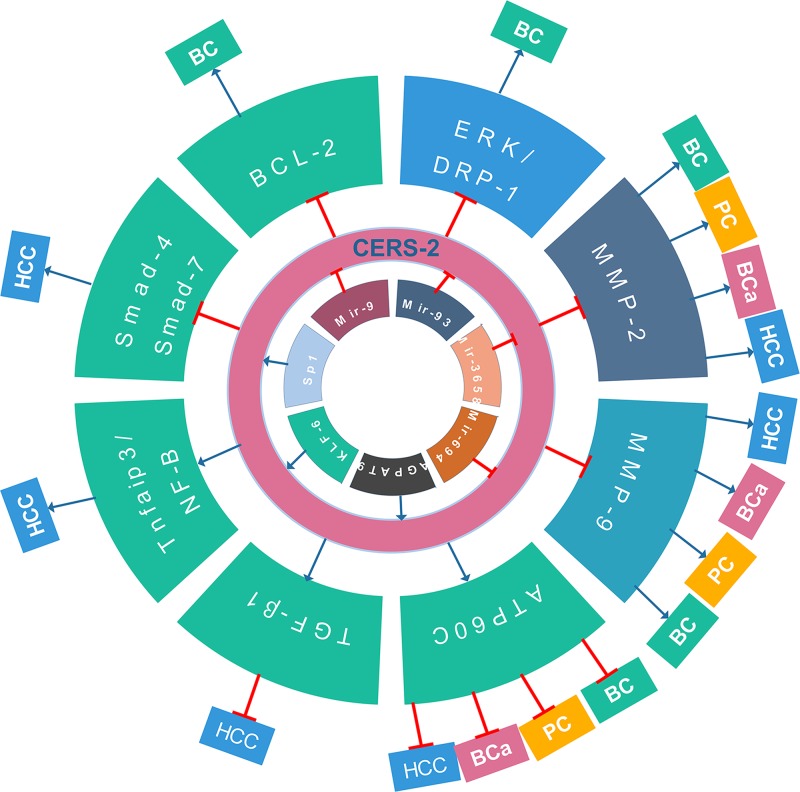
Summary of current research on CerS-2 in bladder cancer (BC), hepatocellular carcinoma (HCC), breast cancer (BCa) and prostatic cancer (PC)

In most of the above-mentioned diseases, CerS-2 levels are down-regulated, and thus recognizing this gene as a tumor suppressor (Table 1). The loss of CerS-2 expression is related with oncogenic properties through the loss of its role as a tumor suppressor. To be detailed, CerS-2 expression absence is significantly related with aggressive tumor behaviors, such as high drug resistance, low differentiation grade, and high recurrence rate, as well as shorter survival duration, including disease-free survival and overall survival. According to experimental studies on animals or *in vitro*, CerS-2 has been proven to be an effective candidate for treatment. Furthermore, CerS-2 is a significant, independent prognostic factor predicting survival in various tumors, suggesting that it can serve as a novel diagnostic and prognostic biomarker. By combining genomic, epigenetic, and expression data to identify clinically significant tumor biomarkers, CerS-2 expression is predicted to be a useful, functionally relevant biomarker (Figure 1). Future studies are still needed to address whether CerS-2 down-regulation is a cause or consequence of the progression of most human cancers. Understanding of the role of CerS-2 in some cancers is still at the initial stage, warranting further investigation.

**Table 1:** Related factors of CerS-2 in diverse human cancers

Type	Expression	Mir-RNA	Related factors	Reference
Bladder Cancer	Low	Mir-9	ATP60C, BCL-2	Wang et al. [9], Zhan et al. [12]
		Mir-93	MMP-9	Liu et al. [10], Zhan et al. [12]
		Mir-3658	MMP-2	Luan et al. [11], Zhan et al. [12]
			ERK/DRP-1	Huang et al. [13]
Hepatocellular carcinoma	Low	Mir-694	ATP60C, Tnfaip3	Lu et al. [27], Gu et al. [28]
			TGF-β1, Smad-4, Smad-7	Chen et al. [23]
Breast Cancer	Low		ATP60C	Fan et al. [33]
			AGPAT9	Fan et al. [37]
			MMP-2, MMP-9	Fan et al. [34], Mei et al. [35]
Prostatic cancer	Low		ATP60C, MMP-2, MMP-9	Xu et al. [42], Xu et al. [43], Zou et al. [44]
			Sp-1, KLF-6	Gong et al. [45]

## Abbreviations

BC: bladder cancer
BCa: breast cancer
CerS-2: *Homo sapiens* ceramide synthase 2
HCC: hepatocellular carcinoma
IR: ionizing radiation
PC: prostate cancer
PCC: pheochromocytomas
